# Post-kala-azar Dermal Leishmaniasis with Mucosal Involvement: An Unusual Case Presentation including Successful Treatment with Miltefosine

**DOI:** 10.3329/jhpn.v31i2.16395

**Published:** 2013-06

**Authors:** Md. A. Salam, Muhammad A. Siddiqui, Shah G. Nabi, Khondaker R.H. Bhaskar, Dinesh Mondal

**Affiliations:** ^1^Rajshahi Medical College, Rajshahi 6000, Bangladesh;; ^2^Communicable Disease Control, Directorate General of Health Services, Mohakhali, Dhaka, Bangladesh;; ^3^icddr,b, Mohakhali, Dhaka 1212, Bangladesh

**Keywords:** Miltefosine, treatment, Mucosal involvement, Post-kala-azar dermal leishmaniasis, Bangladesh

## Abstract

Post-kala-azar dermal leishmaniasis (PKDL) is a dermatologic manifestation that usually occurs after visceral leishmaniasis (VL) caused by *Leishmania donovani.* It is characterized by hypopigmented patches, a macular or maculopapular rash and nodular skin lesions on the body surface. Involvement of the mucosae is very rare and unusual in PKDL. We report a case of PKDL that presented with polymorphic skin lesions, along with involvement of peri-oral mucosa and tongue from an endemic area for kala-azar in Bangladesh. In the absence of a definite past history of kala-azar, a clinical suspicion for PKDL was confirmed by positive rapid serological tests against two recombinant (rK39 and rK28) leishmanial antigens, demonstration of *Leishmania donovani* (LD) body in the slit skin smear, and isolation of promastigotes by culture from a nodular lesion. The patient was treated with oral Miltefosine for three consecutive months and showed significant clinical improvement as demonstrated by a negative slit skin smear at two months after initiation of therapy. We report this case as an unusual presentation of mucosal involvement in PKDL and subsequent treatment success with Miltefosine.

## INTRODUCTION

Post-kala-azar dermal leishmanisis (PKDL), first described by Brahmachari in 1922, is a dermatologic manifestation which usually occurs months to years after resolution of visceral leishmaniasis (VL) caused by *Leishmania donovani* (1). PKDL has also been reported in individuals without a prior history of VL, suggesting subclinical infection (2). Incidence of PKDL after VL varies from 5% to 10% in the Indian Subcontinent while it is about 60% in Sudan (3). Typical clinical manifestations of PKDL include hypopigmented patches, a macular or maculopapular rash, and nodular lesions in people who are otherwise well (2). Mucosal involvement in PKDL is rare (4,5) and till now has not been reported from Bangladesh, a country endemic for kala-azar and PKDL. Unlike kala-azar which is ultimately fatal without treatment, PKDL is not usually associated with systemic illness but it is speculated that patients can remain infectious for years or even decades (6). Further, patients with PKDL represent an important but largely neglected likely reservoir of infection that perpetuates anthroponotic transmission of VL in the Indian Subcontinent. As a consequence, detection and treatment of PKDL is an essential measure toward any attempt at VL eradication. Till now there has been limited information on the burden of PKDL in the VL-endemic regions of Bangladesh. One study found that the prevalence of PKDL was 6 per 10,000 people in a VL-endemic area in Mymensingh, Bangladesh (7). Increasing incidence of PKDL from 1 case per 10,000 person-years in 2002-2004 to 21 cases per 10,000 person-years in 2007 in Bangladesh was observed by Rahman *et al*. (8). The rising PKDL incidence threatens the regional VL elimination initiative and underscores the urgent need for more effective PKDL diagnosis and treatment.

## CASE HISTORY

A 40-year old day-labourer (male) from Godagari subdistrict, a kala-azar-endemic area of Rajshahi district, Bangladesh, presented with gradually-developing hypopigmented patches over different parts of the body for 12-15 years, followed by erythematous papular and nodular lesions over the face, neck, and trunk for the last 8-10 years, along with concomitant involvement of peri-oral mucosa and tongue (Figure 1a and 1b). Associated complaints included change of voice and intermittent difficulty in swallowing for the same duration. He attended the outpatient Dermatology Department of Rajshahi Medical College Hospital (RMCH) and was suspected clinically to be a case of post-kala-azar dermal leishmaniasis (PKDL). He was then referred to the Department of Microbiology, Rajshahi Medical College, for serological and parasitological evaluation. Rapid Immunochromatographic test (ICT) against both rK39 (Kalazar Detect; InBios International, Inc. Seattle, USA) and rK28 (DPP Leishmania, Chembio Diagnostic System, Inc., NY, USA) leishmanial antigens was found to be positive. Leishman-stained slit skin smear (SSS) prepared from a nodular skin lesion at the neck was also found positive for *Leishmania donovani* (LD) bodies (Figure 2). Culture of aspirated fluid from a prominent nodular lesion present on the neck in Novy, MacNeal and Nicolle (NNN) medium overlaid with RPMI 1640 yielded promastigotes after 5 days of incubation at 25 ^0^C. The patient reported fever of about three months duration when he was 9 years old but there was no history of VL diagnosis nor treatment with injectable drugs for that fever. He did not give any family history of kala-azar. On systemic examination, pulse was 78/min, blood pressure 110/70 mmHg, and there was no evidence of anaemia, hepatosplenomegaly, lymphadenopathy or cardiac abnormality. Examination of other systems did not show any abnormalities.

**Figure 1a.**
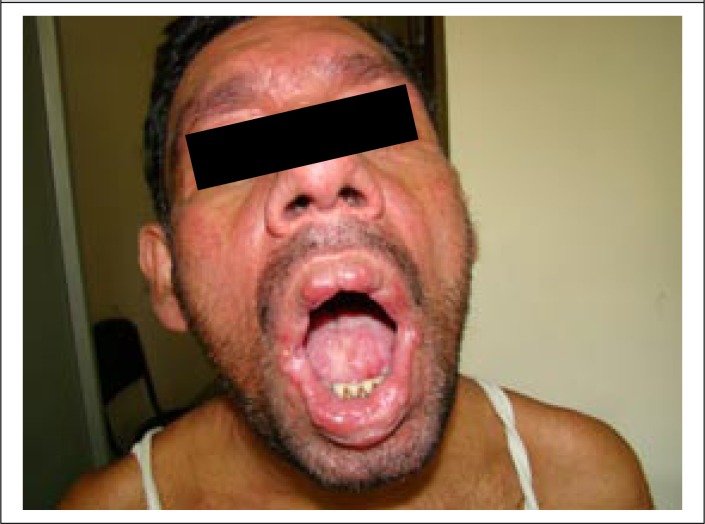
Presentation of PKDL with peri-orificial mucosal and tongue involvement at diagnosis

**Figure 1b.**
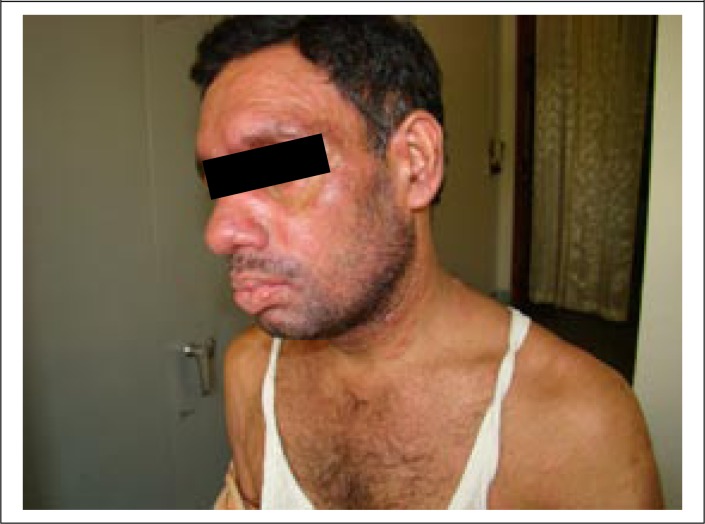
Presentation of PKDL with prominent skin and mucosal lesions at diagnosis

**Figure 2.**
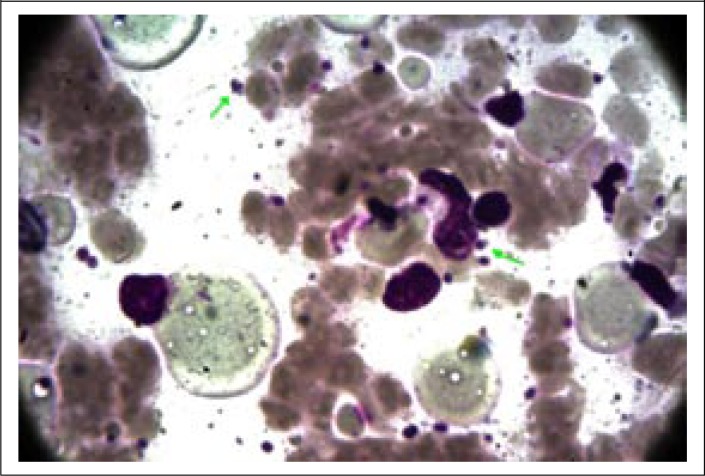
Amastigotes (x 1000) in slit skin smear taken from nodular lesion

Laboratory investigations revealed a total white blood cell count (WBC) of 4,000/mm^3^ (ref. value 4,000-11,000/mm^3^) with 58% neutrophils, 34% lymphocytes, 4% monocytes, and 4% eosinophils, haemoglobin 13.8 g/dL, erythrocyte sedimentation rate (ESR) 70 mm in the first hour, platelet count 240,000/mm^3^, serum glutamate pyruvate transaminase (SGPT) 39 U/L, serum creatinine 97 µmol/L, and random serum glucose 7.8 mmol/L. A serological test for HIV was negative. Ultrasonography of the whole abdomen suggested no pathology; chest radiograph was normal; and electrocardiogram revealed sinus tachycardia. His body-weight was 55 kg.

**Figure 3.**
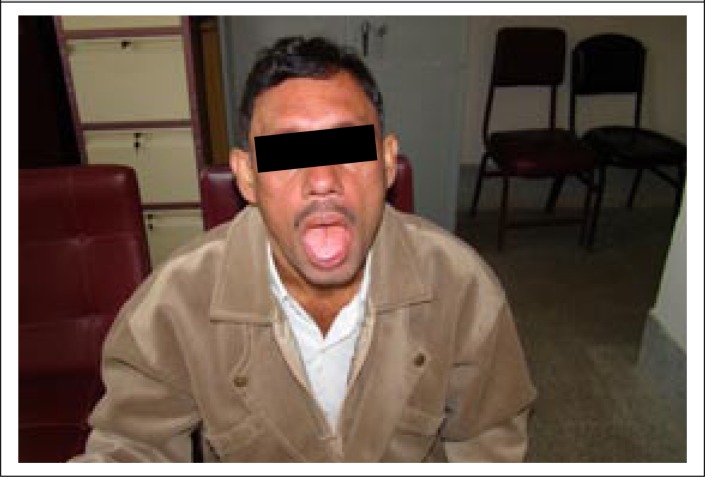
Improvement after 2 months of treatment with Miltefosine

The patient was treated with Miltefosine (50 mg twice daily) for three consecutive months. After 2 months of therapy, follow-up revealed significant clinical improvement of his condition with a total WBC count of 6,500/mm^3^ and a normalized ESR. There was marked regression of the nodular lesions of both body and the tongue while the erythematous lesions were reduced and faded but had not disappeared (Figure 3). Follow-up slit skin smear taken from an erythematous lesion was found negative for LD bodies. Treatment was well-tolerated, and no adverse effects were observed. He was advised to continue the drug for another month (i.e. 3 months in total) and to attend the outpatient department for periodic follow-up. Informed written consent was obtained from the patient before performing the laboratory investigations and also for publication of his photos.

## DISCUSSION

Despite initial suspicions of PKDL, the involvement of the mucosae and the absence of a history of kala-azar made this case a diagnostic dilemma. Eventually, we confirmed a diagnosis of PKDL through both parasitological and serological tests. Until recently, the diagnosis of PKDL was largely based on clinical and epidemiological suspicions. Demonstration of parasites in slit skin smear or by culture of the dermal tissue is considered to be the gold standard but such methods are invasive and less sensitive (58%) because of low parasite burden and, thus, not feasible in field conditions (9). On the contrary, recent availability of rapid serodiagnostic tools, like the rK39, has revolutionized the diagnosis of VL as well as PKDL (sensitivity of around 95% for polymorphic PKDL and 78% for macular variety) (10). As a result, this simple and rapid test has been recommended and is being widely used in detection of both VL and PKDL in the elimination programmes of India, Bangladesh, and Nepal. Although diagnosis by PCR using samples from skin lesions and peripheral blood buffy coat has been found to be promising for PKDL (10), it is still not available as a point-of-care test in VL-endemic areas.

The pathogenesis of PKDL remains unknown. Several factors may be implicated, such as host genetic susceptibility, *Leishmania* species, and the immune state of the host. In fact, PKDL is a unique example of a disease with visceral involvement that evolves after therapy to involve a different organ of the same host, as if the immune response limited the disease to the skin but could not destroy the organisms completely—a failure of the cellular immunity against a dermotropic variant (6). Various rare morphological forms of PKDL, including mucosal, xanthomatous, verrucous, papillomatous, hypertrophic, fibroid, atrophic, and extensive hypopigmentation, have been documented from regions endemic for PKDL (11,12). Lesions of PKDL can also affect the oral mucosa as seen by granulomatous nodules at the angles of mouth, over the dorsum of the tongue, buccal mucosa, or the soft palate (6,13). The mucosal involvement of the case described above was in accordance with such lesions. Oral or peri-oral mucosal involvement in this case may also resemble dermal/mucosal manifestations of conditions, such as drug reactions, secondary syphilitic lesions, oral lichen planus, lupus erythematosus or pemphigus vulgaris. However, for each of these conditions, typical descriptions of skin and/or mucosal lesions are demonstrable, along with a suggestive history. It may be speculated that involvement of mucus membranes may represent a more severe form of PKDL and, perhaps, this can be correlated with a high ESR and borderline leukopenia that were observed in our case. In Bangladesh, standard treatment for PKDL has involved the use of sodium stibogluconate (SSG) for 6 consecutive months, with a 20-day cycle per month, followed by a 10-day interval between two cycles. In June 2012, a VL elimination programme introduced Miltefosine as a first-line drug for the treatment of PKDL. It is expected to be included in an upcoming revision of the National Leishmanisis Treatment Guideline. Although there are no published reports on the therapeutic efficacy of Miltefosine for PKDL in Bangladesh, an Indian study carried out by Ramesh *et al.* has reported excellent therapeutic response of PKDL to Miltefosine (14). Significant clinical improvement and apparent parasitological cure of our case with Miltefosine is very encouraging and consistent with the efficacy observed in the Indian report. Further, return of ESR and blood leukocyte count to normal limits after leishmania-specific treatment also indicates that the high ESR and borderline leukopenia before introduction of treatment were likely due to the disease itself.

### Conclusions

To the best of our knowledge, there has been no reported experience on the treatment of mucosal PKDL with Miltefosine in Bangladesh. Albeit very rare, the involvement of mucosa must also be looked for while examining a clinically-suspected patient of PKDL in endemic regions, regardless of the past history of kala-azar. Considering the reported efficacy of Miltefosine for PKDL observed in an Indian study and taking into account the patients’ compliance and potential toxicities of SSG, we recommend more studies on Miltefosine for treatment of PKDL in Bangladesh.

## ACKNOWLEDGEMENTS

We gratefully acknowledge Dr. Daniel T. Leung, Division of Infectious Disease, Massachusetts General Hospital and Instructor, Department of Medicine, Harvard Medical School, Jackson 504, 55 Fruit Street, Boston, MA 02114, for his contribution in editing the language of this manuscript.
